# Motor Network Plasticity and Low-Frequency Oscillations Abnormalities in Patients with Brain Gliomas: A Functional MRI Study

**DOI:** 10.1371/journal.pone.0096850

**Published:** 2014-05-07

**Authors:** Chen Niu, Ming Zhang, Zhigang Min, Netra Rana, Qiuli Zhang, Xin Liu, Min Li, Pan Lin

**Affiliations:** 1 Department of Medical Imaging, First Affiliated Hospital of Xi'an Jiaotong University, Xi'an, Shaanxi-Province, P. R. China; 2 Institute of Biomedical Engineering, Xi'an Jiaotong University, Xi'an, Shaanxi-Province, P.R. China; University Of Cambridge, United Kingdom

## Abstract

Brain plasticity is often associated with the process of slow-growing tumor formation, which remodels neural organization and optimizes brain network function. In this study, we aimed to investigate whether motor function plasticity would display deficits in patients with slow-growing brain tumors located in or near motor areas, but who were without motor neurological deficits. We used resting-state functional magnetic resonance imaging to probe motor networks in 15 patients with histopathologically confirmed brain gliomas and 15 age-matched healthy controls. All subjects performed a motor task to help identify individual motor activity in the bilateral primary motor cortex (PMC) and supplementary motor area (SMA). Frequency-based analysis at three different frequencies was then used to investigate possible alterations in the power spectral density (PSD) of low-frequency oscillations. For each group, the average PSD was determined for each brain region and a nonparametric test was performed to determine the difference in power between the two groups. Significantly reduced inter-hemispheric functional connectivity between the left and right PMC was observed in patients compared with controls (*P*<0.05). We also found significantly decreased PSD in patients compared to that in controls, in all three frequency bands (low: 0.01–0.02 Hz; middle: 0.02–0.06 Hz; and high: 0.06–0.1 Hz), at three key motor regions. These findings suggest that in asymptomatic patients with brain tumors located in eloquent regions, inter-hemispheric connection may be more vulnerable. A comparison of the two approaches indicated that power spectral analysis is more sensitive than functional connectivity analysis for identifying the neurological abnormalities underlying motor function plasticity induced by slow-growing tumors.

## Introduction

Brain plasticity is the reshaping of the nervous system during routine activities (e.g., learning or memory) or following pathological conditions (e.g., neoplasms or traumatic brain injury). This continuous remodeling process aims to optimize the functioning of brain networks [1]–[3]. Progressive lesions, such as slow-growing tumors, may induce a larger functional reshaping. Many believe that this is the explanation for why neurological deficits do not appear earlier, even though the lesion lies within the so-called eloquent areas [4]. Additionally, the reorganization of functional areas may take place during tumor growth. Therefore, understanding such functional reorganization is not only important for mapping the resection margin, but is also helpful for to predict the functional outcomes of surgery and to prepare for rehabilitation. Yet, the exact neurobiological mechanisms underlying functional plasticity caused by brain tumors remain elusive.

In the last two decades, resting-state functional magnetic resonance imaging (rs-fMRI) has been widely used in the study of both normal subjects [5]–[7] and patients with brain disorders for assessing functional connectivity (FC), which involves analysis of spatially distributed and temporally correlated signals between brain regions. Biswal et al. [5] first reported the presence of spontaneous low-frequency oscillations (LFOs) that were highly synchronous between the right and left primary motor cortex (PMC) at rest, using fMRI. In addition, abnormal FC was found in a wide range of brain disorders including autism [8], [9], Alzheimer's disease [10], attention deficit hyperactivity disorder [11], mild cognitive impairment [12], and schizophrenia [13].

Recently, brain tumor induced network connectivity dysfunction has been proposed in several studies, involving language, sensorimotor, and default-mode network [14]–[18]. However, research on the regional properties of the brain's intrinsic functional dynamics is lacking. Some recent studies have indicated that power spectral density (PSD) analysis is a sensitive method for detecting and characterizing blood oxygen level-dependent (BOLD) signal oscillations. The advantage of this approach is that it can identify the oscillatory dynamics of the BOLD signal across a wider range of frequencies than FC [19], [20]. Recent studies using PSD analysis demonstrated increased high-frequency oscillations within certain pain-related brain regions in several pain diseases [19], [21]. Furthermore, in contrast to FC, PSD analysis can provide valuable information on the regional characteristics of spontaneous changes in the low-frequency fluctuations, as well as of changes in BOLD signal dynamics associated with neural activity. [22]–[25]. A small number of studies show that independent frequency bands are associated with specific brain function [23], [26], [27]. Furthermore, it has been shown that patients with cognitive disorders exhibit frequency-dependent changes in abnormal LFO amplitudes [28], [29]. However, it is still not clear whether any PSD abnormalities are related to specific frequency sub-bands of the LFOs.

Brain tumor infiltration and compression of the cortex and subcortical white matter are thought to result in cortical dysfunction [30], [31]. Accordingly, the brain function and rhythmic oscillations may be altered in the brain regions which show functional connectivity disruption in patients with brain tumors [31]. Furthermore, individual differences in tumor location, histopathology, growth patterns, and brain functional plasticity may induce FC or PSD changes in tumor patients. Little is known about whether patients with brain gliomas show abnormal PSDs across the LFOs bands and whether any LFO sub-bands are especially informative scientifically and diagnostically in these patients.

To address the above issues, in the current study, we limited our patient selection to a single pathology (glioma) with a restricted location (within or close to the PMC) and measured the FC of the motor network using functional connectivity magnetic resonance imaging (fcMRI). We hypothesized that the brain tumor would infiltrate, compress, and destroy motor areas and induce abnormal PSD in motor cortical regions of patients with brain tumors even in those without motor weakness. Specifically, we used rs-fMRI to investigate the possible alteration of PSD in the oscillatory dynamics of the BOLD signal across different frequency bands in patients with brain gliomas, and compared them with those of age- and gender-matched healthy controls. Ultimately, using both fcMRI and power spectral analysis, we examined the relationship between alterations of LFOs and plastic changes in motor FC in patients with brain gliomas to achieve a better understanding of the underlying brain plasticity mechanisms.

## Materials and Methods

### Ethics statement

All subjects were fully informed of the nature of the study and all gave their written consent regarding participation. This study was approved by the local ethical committee of the Xi'an Jiaotong University Institutional Review Board for clinical research.

### Patients and controls

We consecutively evaluated 143 patients with brain tumors using conventional MRI, based on a prospective study design. These patients were registered at the First Affiliated Hospital of Xi'an Jiaotong University between May 2011 and March 2013. After reviewing all conventional MRI scans obtained prior to surgery, as well as the post-surgical pathology results, 15 patients (11 male and 4 female; age range, 27–65 years; mean age, 49.27±10.65 years, all right-handed) with a histopathologically confirmed brain glioma, were selected for the study (Table 1). All subjects had a space-occupying lesion located in the vicinity of the central sulcus (near or within the PMC) recognized based on a previous computed tomography (CT) or MRI examination (with or without contrast medium). Seven patients (46.7%) presented with headache, 5 (33.3%) with seizures, 2 (13.3%) with a history of vomiting, and 1 (6.7%) was asymptomatic and incidentally diagnosed during an imaging study performed for other reason. All patients had normal muscle strength and had no motor weakness according to both the manual muscle testing scale (MTT) and a clinical exam.

**Table 1:** Demographic information and tumor classification for study subjects.

No.	Symptoms	Age (years)	Gender	Tumor location	Tumor type
1	Seizure	42	F	Left Frontal	Astrocytoma (Grade II)
2	Seizure	39	M	Left Parietal	Astrocytoma (Grade II)
3	Headache	45	F	Left Frontal/ Parietal	Astrocytoma (Grade II)
4	Persistent vomiting	27	M	Right Frontal/ Parietal	Oligodendroglioma (Grade II)
5	Seizure	38	M	Right Frontal/ Parietal	Oligodendroglioma (Grade II)
6	Seizure	47	M	Left Frontal	Oligodendroglioma (Grade II)
7	Headache	65	F	Right Parietal	Astrocytoma (Grade II)
8	Headache	61	M	Left Frontal	Astrocytoma (Grade III)
9	Headache	53	M	Left Frontal/ Parietal	Astrocytoma (Grade III)
10	Asymptomatic	41	M	Left Parietal	Oligodendroglioma (Grade II)
11	Headache	63	M	Right Parietal	Astrocytoma (Grade III)
12	Vomiting	56	M	Left Frontal	Astrocytoma glioma (Grade III)
13	Seizure	51	M	Left Frontal/ Temporal	Astrocytoma glioma (Grade II)
14	Headache	55	F	Right Frontal/ Parietal	Astrocytoma glioma (Grade II)
15	Headache	56	M	Left Frontal/ Parietal	Astrocytoma glioma (Grade III)

M =  male; F =  female.

Additionally, 15 healthy volunteers, (11 male and 4 female; age range, 25–60 years; mean age, 46.07±9.42 years, all right-handed), were recruited as a control group. The eligibility criteria for healthy volunteers consisted of the absence of any pre-existing or presenting abnormal neurological conditions, and the volunteers underwent structural MRI scanning without administration of contrast medium prior to undergoing fMRI.

### Data acquisition

All images were acquired using a 3.0T whole-body scanner (GE Signa HDxt, Milwaukee, WI, USA) equipped with an 8-channel head receiver coil. Head movement was restricted using a pillow and foam, and earplugs were used to minimize scanner noise and maximize patient comfort.

#### Anatomical imaging

A three-dimensional T1-weighted fast spoiled gradient echo (FSPGR) sequence covering the whole brain was performed to coregister functional data and define regions of interest (ROI) (time of repetition [TR] /time of echo [TE] /flip angle [α]  = 10.8 ms/4.8 ms/15°; field of view  = 256 mm; matrix  = 256×256; slice thickness  = 1 mm; no gap; voxel size  = 1×1×1 mm^3^; 150 axial plane).

#### Functional imaging

BOLD functional images were acquired by means of a T2*-weighted single-shot gradient-echo-planar-imaging sequence with the following parameters: TR = 2500 ms; TE = 40 ms; α = 90°; field of view  = 256 mm; acquired matrix  = 64×64; slice thickness  = 3 mm; voxel size  = 3.75×3.75×3 mm^3^; 47 slices; no gap. A total of 150 functional volumes were acquired.

### fMRI motor task design

A “block” design sequence (ABAB) was used for the motor task, with six 30-s rest periods (A) alternated with five 30-s periods of visual cues for hand movement (B). All patients and healthy controls were instructed to repetitively open and close both hands in response to each flash used as a visual cue. All subjects were trained in the task and were observed before performing the experiment to ensure their understanding and ability to comply with the protocol. No cue was supplied during rest periods. The experimental stimuli were presented using E-Prime Version 2.0 (Psychology Software Tools, Pittsburgh, PA, USA), transmitted via a liquid crystal display projector, and viewed through a mirror placed above the subject's head.

We performed the resting-state scanning prior to the motor task scanning. For the task-free functional experiment, all participants were instructed to relax and remain calm with their eyes open. Subjects were instructed to not think of anything in particular without falling asleep.

### Data analysis

#### Motor task fMRI data analysis

The motor task fMRI dataset was analyzed with FSL software (www.fmrib.ox.ac.uk/fsl/). The first four scans were discarded, and then motion correction was applied using FLIRT (MCFLIRT). Spatial smoothing was performed using a 6-mm full-width-half-maximum (FWHM) Gaussian kernel to reduce noise. The functional connectivity MRI images were filtered with a high-pass filter. The functional images were normalized to the MNI152 standard brain space through their structural images. General linear model (GLM) analysis was carried out using FSL FEAT. Z statistic images were thresholded using clusters determined by Z>2.3, a corrected cluster significance threshold of *P*<0.05. All 3 regions including the left PMC (LPMC), right PMC (RPMC), and supplementary motor area (SMA) could be detected via a task-evoked BOLD response. As a result, we extracted the maximum activation mapping to define the key motor regions for resting-state inter-regional functional analysis.

#### Inter- regional functional connectivity analysis

The rs-fMRI analysis was performed using AFNI (Cox, 1996) and FSL software (www.fmrib.ox.ac.uk/fsl/). Pre-processing consisted of motion correction, temporal band-pass filtering (0.008 Hz<f<0.1 Hz), spatial normalization to standard Talairach space and spatial smoothing (Gaussian, FWHM 6 mm). Several sources of nuisance covariates (6 head motion parameters, signal from the white matter and the CSF) were eliminated using linear regression. The three key motor network regions (LPMC, RPMC, and SMA) were selected based on motor task functional mapping for each subject, and defined as a spherical region with a radius of 10 mm. Mean time series from three Regions of interest (ROIs) were estimated by averaging the time series of all voxels in a region. In the present study, Pearson's correlation coefficients were computed between each pair of brain regions for each subject. For further analysis, a Fisher's r-to-z transformation was applied to improve the normality of the correlation coefficients. In general, normal brain areas can be infiltrated and damaged by tumors. As a previous study indicated that functional zones could shift in the presence of neoplastic disease [4], we suspected the possibility that the spatial distribution of functional activation areas between patients and healthy subjects may be inconsistent. In order to test the reliability of our results, we further analyze our fMRI data based on different ROIs (6 mm and 8 mm).

#### Spectral power analysis

Spectral analysis was performed using home-made Matlab (The MathWorks, 2010) code. We derived the PSD estimation using a direct fast Fourier transform method for each subject's motor cortex region resting-state network (RSN) time series. Our specific interest was to compare the power spectral density within this frequency domain between patients and healthy controls, for measurements in the motor cortex. Previous studies have divided the full frequency band (0–0.25 Hz) into following sub-bands: slow-5 (0.01–0.027 Hz), slow-4 (0.027–0.073 Hz), slow-3 (0.073–0.198 Hz), and slow-2 (0.198–0.25 Hz) [23], [26]. These can be further subdivided into additional sub-bands to better reflect the neural origins of the signal sources [23]. Cordes et al suggested that the respiratory and aliased cardiac signals fall into the range of slow 2–3 [7], while the oscillatory signals upon which resting-state FC is primarily fall within slow 4–5 [32], [33]. To simplify this, we divided the low frequency (0.01–0.08 Hz) band into three sub-bands, including “low-band” (0.01–0.02 Hz), “middle-band” (0.02–0.06 Hz), and “high-band” (0.06–0.08 Hz). For each patient with tumor and normal subject, the predominant power spectral density of each motor cortical region (computed from the resting-state time-course) was estimated in each sub-band.

#### Statistical analysis

All statistical calculations were performed by using Statistical Package for the Social Sciences, Version 16.0 (SPSS, Chicago, Illinois). Shapiro-Wilks test was used to assess the normality of all data. Group differences in FC and PSD analysis were compared by using non-parametric Mann-Whitney U tests. For all group-level statistical significance threshold was set at *P*<0.05, two tailed.

## Results

### Functional Magnetic Resonance Imaging

**Figure 1:**
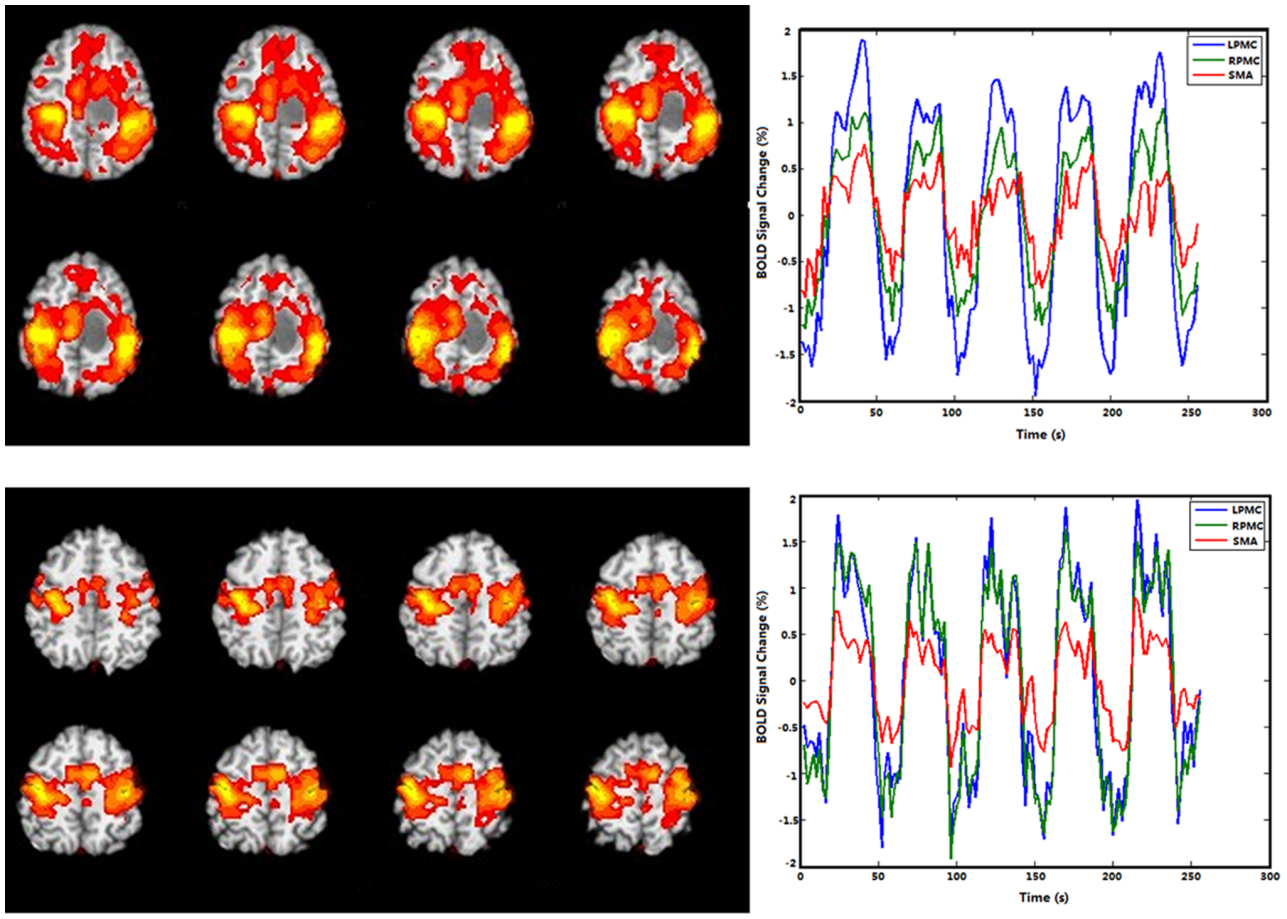
Example of BOLD functional magnetic resonance imaging activation maps and BOLD signal time courses in the LPMC, RPMC and SMA during a motor task in a single patient with glioma and a healthy control subject. BOLD, blood oxygen level dependent; LPMC, left primary motor cortex; RPMC, right primary motor cortex; SMA, supplementary motor area.

Both healthy controls and patients with brain tumors underwent resting-state and task-based fMRI scans (Table 1). As shown in Figure 1, the BOLD fMRI activation map and BOLD signal time course of a single tumor patient differs dramatically from that of a control. The reorganization of functional areas could have been induced by the lesion in patients with gliomas. In order to avoid the possible shift of functional areas caused by the tumor, three key motor areas were selected based on the activation maps of the LPMC, RPMC, and SMA associated with motor task stimuli and identified by the GLM analysis. Full details of this analysis strategy are described in the Materials and Methods section. We present our data processing steps in the form of a flow chart in Figure S1.

### Functional connectivity within the motor network

**Figure 2:**
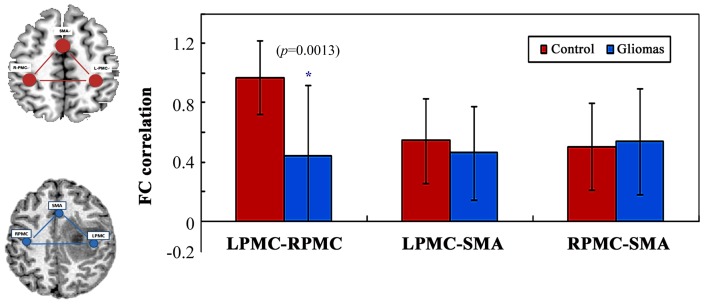
Group differences in the functional connectivity of the motor network between patients with brain gliomas and healthy controls. Error bars represent standard error of the mean. A blue asterisk indicates significant differences between groups (z = −3.215, *P* = 0.001, Mann-Whitney U test). LPMC, left primary motor cortex; RPMC, right primary motor cortex; SMA, supplementary motor area.

The Pearson's correlation coefficients of inter-regional FC within the motor network were computed between each pair of brain regions (RPMC, LPMC, and SMA) for each subject. FC of control and patient groups are shown in Figure 2. We compared the averaged values of FC within ROIs of the 2 hemispheres, patients showed a statistically significant reduction in the connectivity of the LPMC-RPMC (z = −3. 09, *P* = 0.002, Mann-Whitney U test) compared to controls. In contrast, FC analysis of the LPMC-SMA and RPMC-SMA showed no significant differences between the two groups. (LPMC-SMA: z = −0.892, *P* = 0.373, Mann-Whitney U test; RPMC-SMA: z = −0.145, *P* = 0.885, Mann-Whitney U test; respectively). In order to test the consistency and reliability of the results, different size of ROIs (10 mm, 8 mm and 6 mm) were used in the analysis, all of which generated similar results (see Figure S2).

### Changes in PSD

**Figure 3:**
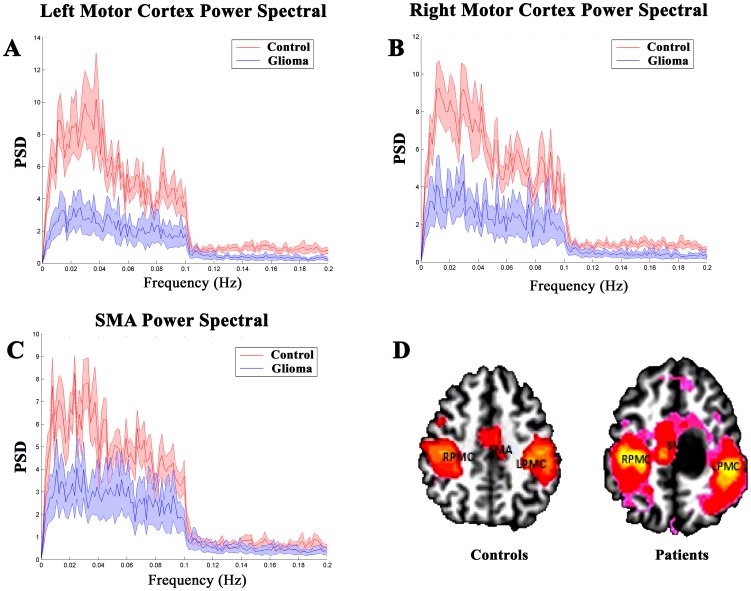
The power spectral density (PSD). A, B, and C show the group mean PSD in the LPMC, RPMC and SMA between healthy subjects (red traces) and patients with brain gliomas (blue traces). D, The localization of three key motor regions. In patients with brain gliomas, the PSDs in the LPMC, RPMC, and SMA are significantly lower than the PSDs of healthy controls (*P*<0.05). LPMC, left primary motor cortex; RPMC, right motor cortex; SMA, supplementary motor area.

The PSDs of BOLD oscillations in the low-frequency band (0∼0.1 Hz) within the LPMC, RPMC, and SMA of controls and patients are shown in Figure 3. Our results demonstrated a remarkable PSD decrease in patients compared to in controls in this range, as shown by the difference between the mean PSD of controls (red traces) and that of patients (blue traces) in the 0–0.1 Hz frequency band within each key motor region. To further test the consistency and reliability of the results, PSD values in ROIs of different sizes (10 mm, 8 mm, and 6 mm) were also calculated and similar results were achieved (see Figure S3).

**Figure 4:**
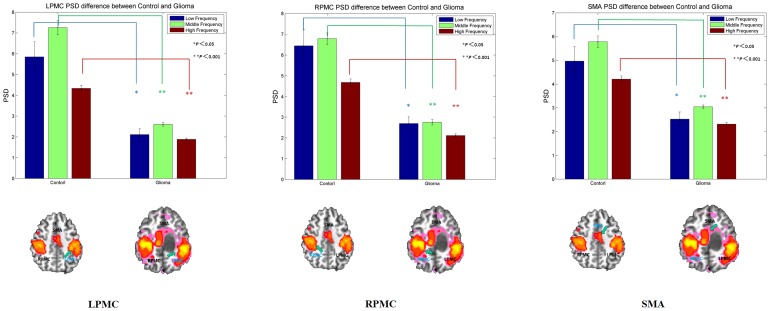
Bar graphs show the PSD in the three non-overlapping frequency bands for the three key regions of the motor network in healthy subjects and patients with brain gliomas. The sub-divisions of the low-frequency band were: low, 0.01–0.02 Hz; middle, 0.02–0.06 Hz; and high, 0.06–0.1 Hz. Patients with brain tumors show significant PSD decreases in all bands in all three key motor cortical regions (LPMC, RPMC, and SMA) (*P<0.05; ** *P*<0.001). PSD, power spectral density; LPMC, left primary motor cortex; RPMC, right motor cortex; SMA, supplementary motor area.

The PSDs of the BOLD oscillation in three frequency bands were estimated for controls and patients with gliomas in the three key motor regions (LPMC, RPMC, and SMA). For each of the frequency bands in the three key regions, we observed a significant decrease in PSD in patients compared to in controls (*P*<0.05, Mann-Whitney U test), as shown in Figure 4 and Table 2. Again, when we used different ROI sizes (10 mm, 8 mm and 6 mm) in repeated analyses to test the consistency of our results, findings were consistent (see Figure S4).

**Table 2:** Statistical comparisons of power spectral density in different brain regions between groups.

PSD	Healthy Controls (N = 15)		Patients (N = 15)		Mann-Whitney U test
	Mean	SD	Mean	SD	P value
**LPMC**					
Low-frequency band*	5.85	2.61	2.11	1.00	*P* = 1. 00×10^−3^
Middle-frequency band**	7.26	1.81	2.60	0.46	*P* = 2. 86×10^−10^
High-frequency band**	4.34	0.69	1.88	0.28	*P* = 1. 33×10^−9^
**RPMC**					
Low-frequency band*	6.45	2.83	2.69	1.15	*P* = 1. 00×10^−3^
Middle-frequency band**	6.78	1.48	2.75	0.67	*P* = 2. 87×10^−10^
High-frequency band**	4.68	0.86	2.11	0.43	*P* = 1. 50×10^−9^
**SMA**					
Low-frequency band*	4.97	2.21	2.53	1.07	*P* = 2. 00×10^−3^
Middle-frequency band**	5.78	1.25	3.04	0.39	*P* = 4. 47×10^−10^
High-frequency band**	4.21	0.67	2.31	0.38	*P* = 1. 33×10^−9^

*implies significant group difference at *P*<0.05;

**implies significant group difference at *P*<0.001. PSD, power spectral density; SD, standard deviation; LPMC, left primary motor cortex; RPMC, right motor cortex; SMA, supplementary motor area. Group-differences were tested using SPSS software.

## Discussion

In this study, we employed FC and power spectral analyses to explore possible changes in the motor network of patients with gliomas. Our results showed a significant difference in the inter-regional FC of the LPMC-RPMC between patients and controls. In addition, patients with brain tumor exhibited abnormal amplitudes of low-frequency fluctuation activity during the resting state, and a significant decrease in PSD within three key motor cortical regions (LPMC, RPMC, and SMA) was found. These results suggest that the low-frequency brain oscillation changes in patients with brain tumors, even in the absence of motor deficits. This finding also indicates that power spectral analysis is more sensitive at detecting the underlying neural mechanism abnormalities during slow-growing tumor-induced brain motor plasticity, and provides a novel insight for explaining how abnormal oscillations might influence brain plasticity. Lastly, this study explores the underlying relationship linking brain plasticity to the LFOs.

### Changes in resting-state functional connectivity within the motor cortex: relationship with motor function plasticity

For patients with brain tumors, brain plasticity plays an important role in motor and language areas. Moreover, brain reorganization is thought to explain why slow infiltrative low-grade gliomas near or in eloquent motor or language areas often do not induce detectable neurological deficits [34]. A number of studies have indicated that many brain diseases may produce a disruption of the normal architecture of the brain by inducing dysfunctional communication between neural networks [35], [36]. According to this view, FC is useful for investigating communication within and between cortical networks. The current study reveals a remarkable difference in motor FC between the LPMC and RPMC in patients with brain tumors compared to in healthy controls, rather than a disruption in connectivity between the SMA and bilateral PMC. Consistent with a previous study [15], this finding confirms that long-distance connections, especially between hemispheres, are particularly vulnerable to damage. The presence of inter-hemispheric plasticity has been established in early brain lesions, while lesions occurring later in life show intra-hemispheric reorganization [37], [38]. In addition, a hierarchically organized model, proposed by Duffau et al [34] explains the sensorimotor and language plasticity mechanisms in slow-growing low grade gliomas (LGG). Initially, intrinsic reorganization within injured areas occurs, and the perilesional structures play a major role in the functional compensation. However, if this reshaping is not sufficient, other regions are recruited to reorganize the functional network, starting with the ipsilateral hemisphere (remote to the damaged area) followed by the contralateral hemisphere [4], [39]. Therefore, we speculate that the reduced inter-hemispheric FC observed in our patients was probably caused by the recruitment of compensating areas from the brain regions surrounding the slow-growing gliomas.

Interestingly, another recent study conducted by Otten et al. using rs-fMRI to investigate sixteen patients with brain neoplasms without motor weakness, reported no significant difference in the motor network connectivity of patients compared to that in controls [15]. However, the key differences include the large degree of heterogeneity in the pathological type and/or brain tumor location used in their study, as well as use of a different analytic method. Since it has been speculated that tumor type, growth pattern, and tumor locations can affect the FC of brain networks [14], [16], the study design of Otten et al may have introduced confounding information via its relatively broad sample size whereas this study concerned a single population of slow-growing histopathologically confirmed gliomas. Therefore the discovery of the eloquent area induced specifically by the mass effect in the gliomas population remains a novel discovery [4]

### Changes in PSD within the motor cortex: relationship with motor function plasticity

In addition to changes in FC within the motor network, our study has demonstrated that patients with gliomas exhibit a decreased PSD in low-frequency bands in each of the three key motor regions during the resting state. This observation provides direct evidence for the specificity of BOLD low-frequency changes in patients with brain tumors.

Previous studies have examined spontaneous LFO activities within the specific frequency band of 0.01–0.1 Hz, because this frequency band is hypothesized to be linked primarily to neural activity [28], [40]–[42]. Additionally, several groups have suggested that a shift in LFOs is associated with some brain-related diseases [28], [29], [43]. These results suggest that changes in the brain's oscillatory dynamics may provide us with novel insights into the neural mechanisms underlying motor functional plasticity induced by brain gliomas. In addition to frequency-specific neural fluctuations, accumulating evidence suggests a possible relationship between PSD and regional spontaneous neural activity and cerebral metabolic rate [44], [45]. Some recent studies have suggested that spontaneous fluctuations in brain activity are concentrated in specific frequency bands [28], [46]. Although the physiological origin and specific functions of various frequency bands remain to be clarified, Zuo et al. [23] subdivided the power spectrum of spontaneous BOLD fluctuations into four different slow-frequency ranges (slow-5, 0.01–0.027 Hz; slow-4, 0.027–0.073 Hz; slow-3, 0.073–0.198 Hz;, and slow-2, 0.198–0.25 Hz). The slow-4 oscillation was most prominent in the thalamus, basal ganglia, and sensorimotor regions, while slow-5 was more prominent in the ventromedial cortical areas. Slow-4 was the most reliable sub-band, with a more widespread spatial distribution of reliable voxels. Based on these observations, we further speculate that decreased PSD in low-frequency bands may indirectly reflect decreased local neural activity caused by tumor growth in the abovementioned motor regions.

Krings et al. [47] also observed a loss of signal intensity in lesions near the tumor, which may be related to tumor-induced hemodynamic changes or to a loss of active neurons. Furthermore, Hou et al. [48] suggested that physiological and biological changes caused by a tumor, such as neovascularization, mass effect, and edema, may alter the blood flow or induce a neurovascular uncoupling effect, thereby changing the hemodynamic responses to activations in functional areas. Ulmer et al. [49] have further suggested that lesion-induced neurovascular uncoupling could cause reduced fMRI signals in the perilesional eloquent cortex, in conjunction with normal or increased activity in homologous brain regions. This may stimulate hemispheric dominance and lesion-induced homotopic cortical reorganization. Taken together, these findings indicate that brain tumors may alter the brain microenvironment, possibly leading to hemodynamic changes and neurovascular uncoupling effects that ultimately result in a dysfunction of processes mediated by LFOs. Our findings provide further insight into the relationship between LFOs and brain function plasticity. It is possible that the dysfunction in LFOs within the motor network observed in this study could also be used in evaluating brain function for presurgical planning or postsurgical assessment.

### Limitations and future perspectives

A few limitations need to be addressed in the interpretation of the results of our study.

#### Sample selection limitations

Although our results are encouraging, the current study is limited by the relatively small sample size; thus, statistical power is of potential concern. Future work should endeavor to increase the sample size so that brain network changes caused by different pathological types of brain tumors may be examined. In addition, in the present study, we only compared patients with gliomas and healthy controls. In our future studies, we intend to increase the sample size and select different histological types of tumor (such as meningioma or metastases) near or within the PMC to constitute an additional control group. This will help to determine whether the observed FC and PSD changes are glioma-specific or a consequence of brain tissue compression/edema.

#### Analysis method limitations

In our study, we observed a potential association between the PSD and functional reorganization in patients with brain gliomas. PSD analysis provides useful frequency information to characterize sensorimotor signal changes induced by brain gliomas. Recently, the frequency-based approach has been used to analyze sensorimotor function. These results indicated that brain low-frequency oscillation is associated with brain function [22], [50]. Although the PSD differences between the patient and control groups were observed in the sensorimotor network, the small sample size means that our results must be treated with caution. Furthermore, only a small number of studies have employed the PSD method to analyze brain function, and its effectiveness needs to be further confirmed by future studies. In addition, other factors could contribute to the changes in PSD. Some studies suggest that the low-frequency fluctuation signal PSD is associated with neurovascular coupling. In particular, a recent cerebrovascular reactivity study showed that neurovascular uncoupling occurs in patients with low grade gliomas [51]. A potential explanation is that neurovascular uncoupling might be linked to brain motor function reorganization. Thus, these results could be helpful for understanding why a PSD shift occurs in patients with gliomas. Despite the interesting finding in our study, the neural basis of the low frequency fluctuation signal PSD deficit induced by brain tumors remains unclear and requires further investigation.

Furthermore, a study performed using only FC and frequency-based analytical approaches is not sufficient to understand the brain plasticity induced by brain tumors. The combination of diffusion tensor imaging and fMRI may provide valuable structure and function information for enhancing our understanding of this issue. Although our study subjects had normal muscle strength and no motor weakness, they were not scored using a behavioral testing method. Future studies should focus on investigating the relationship between LFO signal PSD and motor behavioral performance.

## Conclusions

In summary, we used two different approaches to investigate the motor network plasticity in patients with brain gliomas. First, our results showed abnormal LFOs and dysfunction of inter-hemispheric FC in patients with gliomas without motor deficits. The clinical pre-symptomatic period could be the result of brain plasticity and the reorganization of the eloquent cortex induced by a slow-growing tumor. Secondly, according to our results, the frequency-based analysis may be more sensitive at detecting abnormal LFOs compared to traditional functional connectivity analysis. Emerging evidence suggests that frequency-based analysis is a good indicator of regional neural activity and cerebral metabolic rates in the resting state. Therefore, the frequency-based analysis may provides an important preoperative evaluation of the functionality of brain tissue surrounding the eloquent areas. This study further indicates that frequency-based analysis may have significant potential for addressing other clinical diseases related to abnormal LFOs.

## Supporting Information

Figure S1 **A flow chart of data processing steps.** GLM, general linear model; FC, functional connectivity; ROI, region of interest; PSD, power spectral density.
(TIF)

Figure S2 **Comparison of the functional connectivity of patients and healthy controls using different ROI size.** Group differences in the functional connectivity of the motor network between patients with brain gliomas and healthy controls. Different ROIs (10 mm, 8 mm, and 6 mm) were used, which generate similar results. Error bars represent standard error of the mean. Asterisk indicates significant differences when compared to the control group (P<0.05, Mann-Whitney U test). LPMC, left primary motor cortex; RPMC, right motor cortex; SMA, supplementary motor area.
(TIF)

Figure S3 **The power spectral density of patients and healthy controls using different ROI size.** Power spectral density (PSD) computed using different sizes of ROI (10 mm, 8 mm, and 6 mm). The mean PSD of the left and right PMC between healthy subjects (red traces) and patients with brain gliomas (blue traces) and the group mean PSD of SMA are included. In patients with brain gliomas, the PSDs in the LPMC, RPMC, and SMA are significantly lower than the PSDs of healthy controls (P<0.05, Mann-Whitney U test). LPMC, left primary motor cortex; RPMC, right motor cortex; SMA, supplementary motor area.
(TIF)

Figure S4 **Comparison of the power spectral density of patients and healthy controls in three frequency bands using different ROI size.** Bar graphs show the PSD in the 3 non-overlapping frequency bands for the 3 key regions of motor network in healthy subjects and patients with brain gliomas. Different ROIs (10 mm, 8 mm, and 6 mm) were used, which generated similar results. The sub-divided low-frequency band (low, 0.01–0.02 Hz; middle, 0.02–0.06 Hz; and high, 0.06–0.1 Hz). Patients with brain tumors show a significant decrease in PSD in 3 key motor cortical regions (LPMC, RPMC, and SMA) (*, P<0.05; **, P<0.001).
(TIF)
