# Correction: Motor Network Plasticity and Low-Frequency Oscillations Abnormalities in Patients with Brain Gliomas: A Functional MRI Study

**DOI:** 10.1371/journal.pone.0105134

**Published:** 2014-08-05

**Authors:** The PLOS ONE Staff

There is an error in affiliation 2 for authors Xin Liu and Pan Lin. Affiliation 2 should be: Key Laboratory of Biomedical Information Engineering of Education Ministry, Institute of Biomedical Engineering, Xi'an Jiaotong University, Xi'an, Shaanxi-Province, P. R. China
