# Supplementary figures and images for: Motor Network Plasticity and Low-Frequency Oscillations Abnormalities in Patients with Brain Gliomas: A Functional MRI Study
Source: PLoS One. 2014 May 7;9(5):e96850. doi: 10.1371/journal.pone.0096850 (PMC4013133; doi:10.1371/journal.pone.0096850)

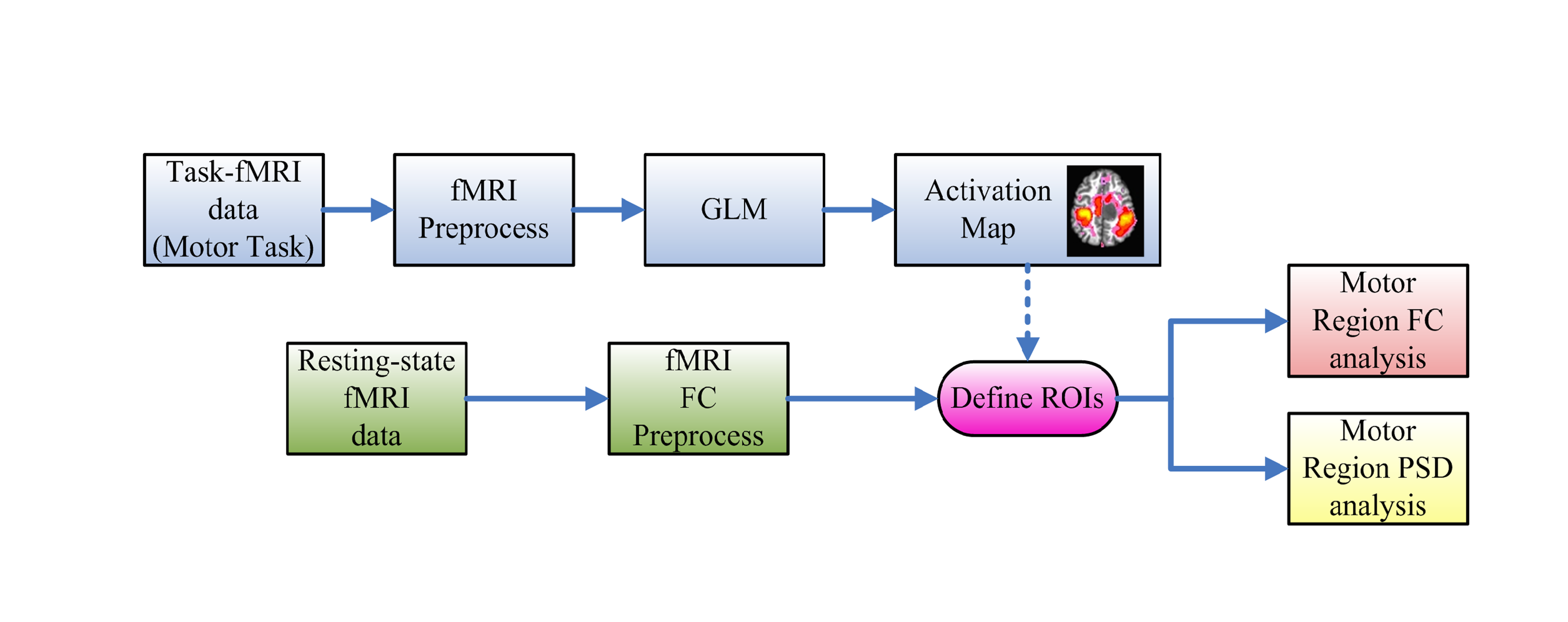

Supplement: Figure S1 — A flow chart of data processing steps. GLM, general linear model; FC, functional connectivity; ROI, region of interest; PSD, power spectral density. (TIF) [file pone.0096850.s001.tif]

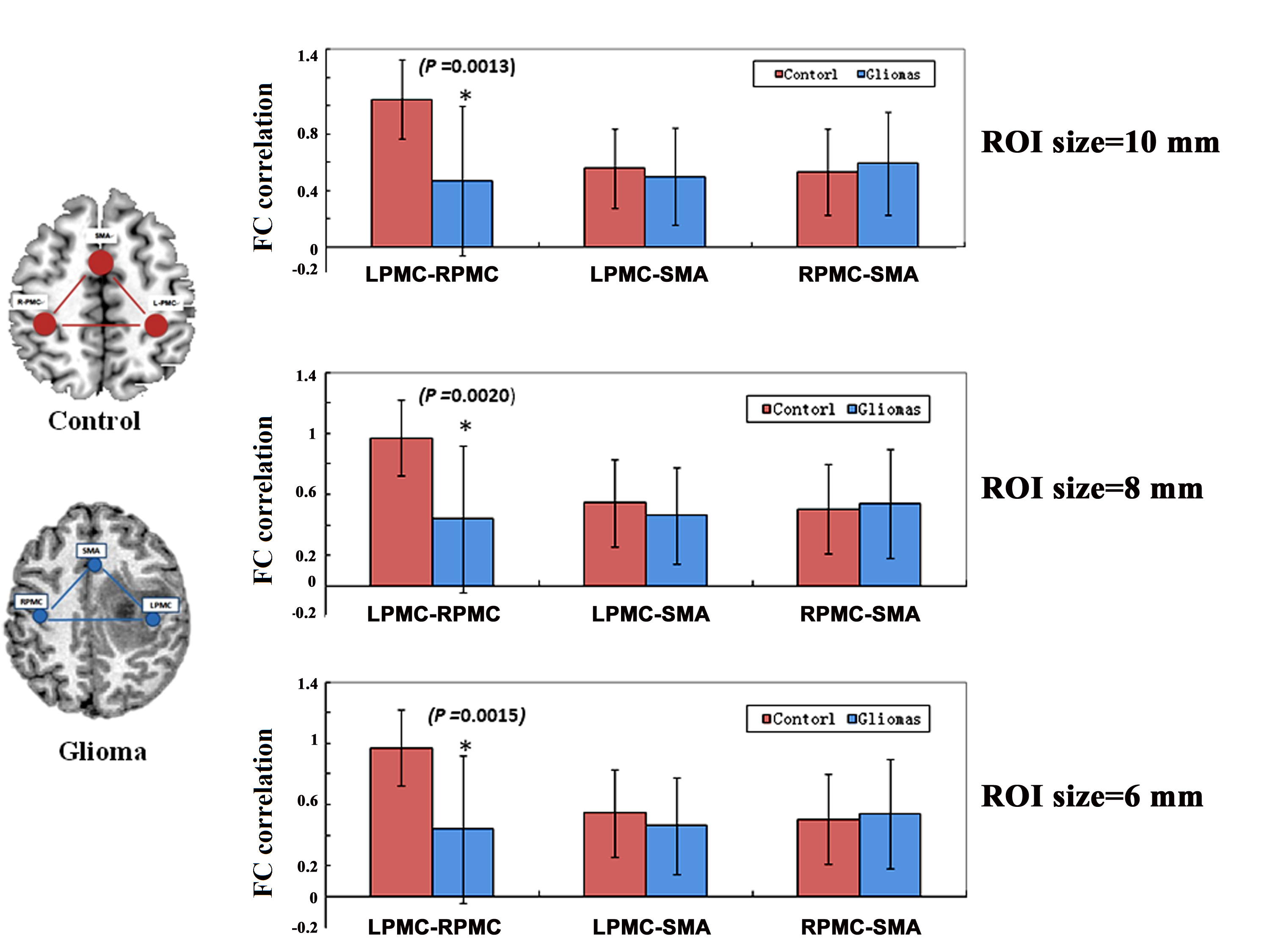

Supplement: Figure S2 — Comparison of the functional connectivity of patients and healthy controls using different ROI size. Group differences in the functional connectivity of the motor network between patients with brain gliomas and healthy controls. Different ROIs (10 mm, 8 mm, and 6 mm) were used, which generate similar results. Error bars represent standard error of the mean. Asterisk indicates significant differences when compared to the control group (P<0.05, Mann-Whitney U test). LPMC, left primary motor cortex; RPMC, right motor cortex; SMA, supplementary motor area. (TIF) [file pone.0096850.s002.tif]

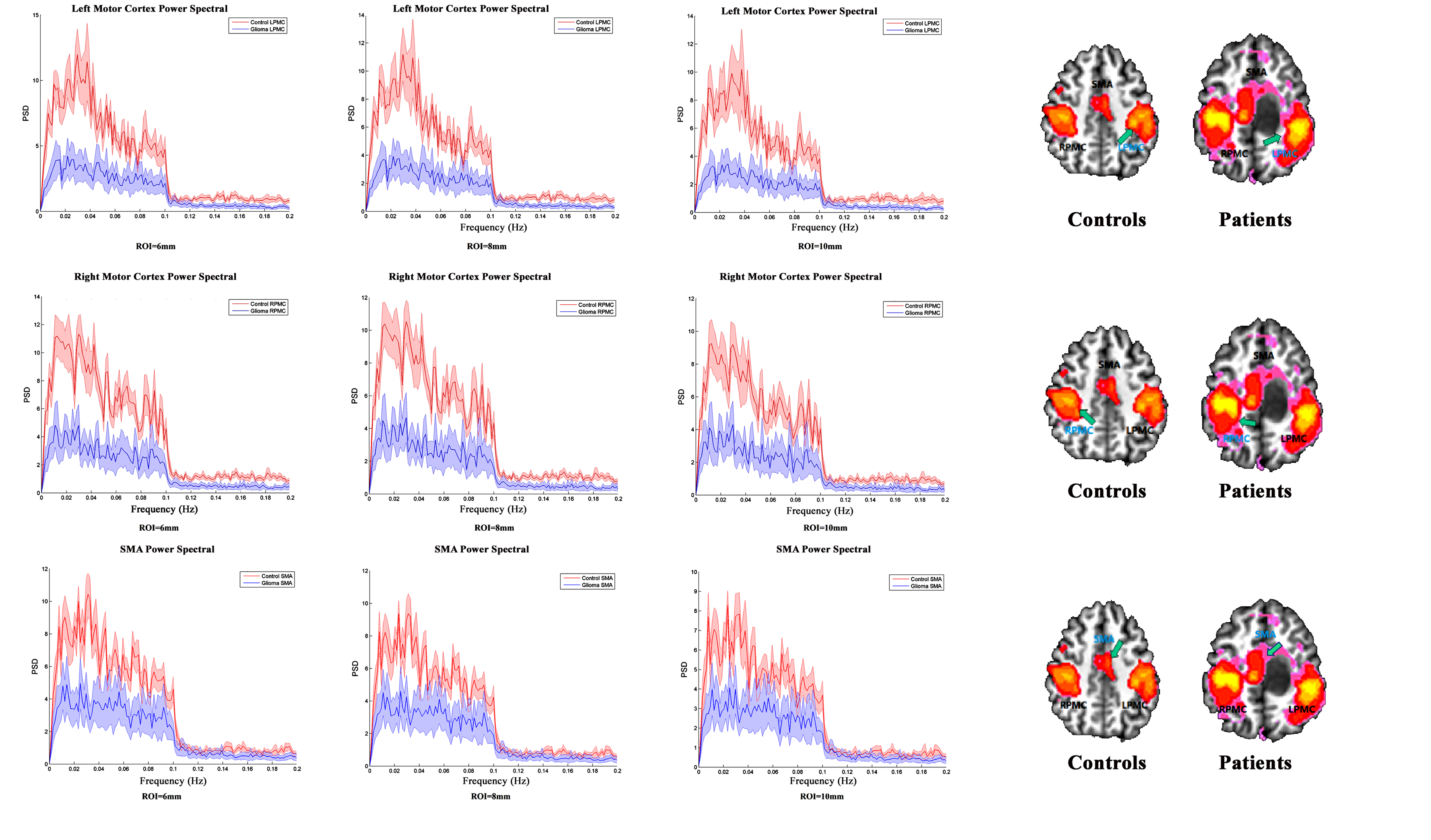

Supplement: Figure S3 — The power spectral density of patients and healthy controls using different ROI size. Power spectral density (PSD) computed using different sizes of ROI (10 mm, 8 mm, and 6 mm). The mean PSD of the left and right PMC between healthy subjects (red traces) and patients with brain gliomas (blue traces) and the group mean PSD of SMA are included. In patients with brain gliomas, the PSDs in the LPMC, RPMC, and SMA are significantly lower than the PSDs of healthy controls (P<0.05, Mann-Whitney U test). LPMC, left primary motor cortex; RPMC, right motor cortex; SMA, supplementary motor area. (TIF) [file pone.0096850.s003.tif]

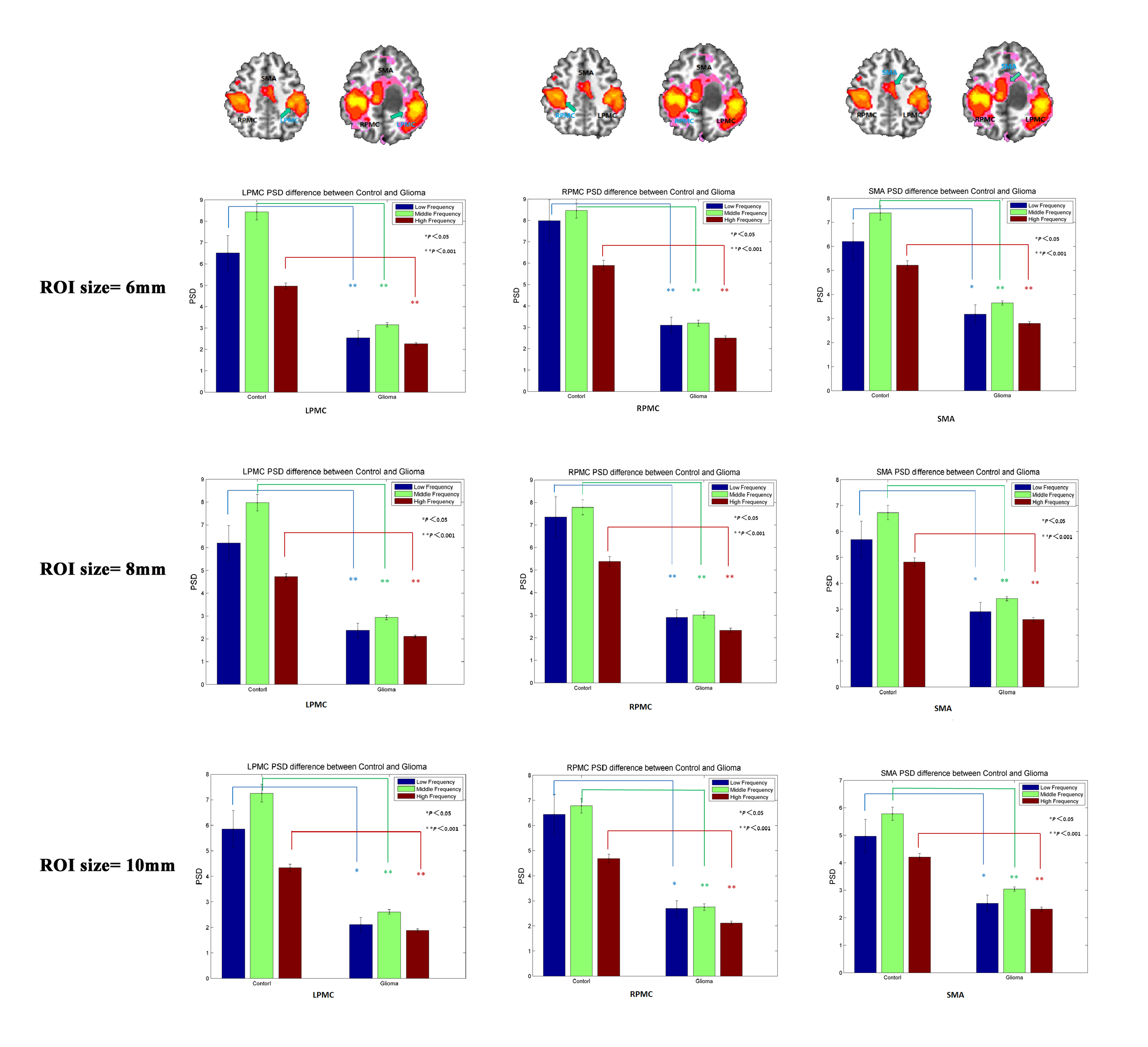

Supplement: Figure S4 — Comparison of the power spectral density of patients and healthy controls in three frequency bands using different ROI size. Bar graphs show the PSD in the 3 non-overlapping frequency bands for the 3 key regions of motor network in healthy subjects and patients with brain gliomas. Different ROIs (10 mm, 8 mm, and 6 mm) were used, which generated similar results. The sub-divided low-frequency band (low, 0.01–0.02 Hz; middle, 0.02–0.06 Hz; and high, 0.06–0.1 Hz). Patients with brain tumors show a significant decrease in PSD in 3 key motor cortical regions (LPMC, RPMC, and SMA) (*, P<0.05; **, P<0.001). (TIF) [file pone.0096850.s004.tif]
